# Bridging the Gap From Proteomics Technology to Clinical Application: Highlights From the 68th Benzon Foundation Symposium

**DOI:** 10.1016/j.mcpro.2024.100877

**Published:** 2024-11-09

**Authors:** Vincent Albrecht, Johannes Müller-Reif, Thierry M. Nordmann, Andreas Mund, Lisa Schweizer, Philipp E. Geyer, Lili Niu, Juanjuan Wang, Frederik Post, Marc Oeller, Andreas Metousis, Annelaura Bach Nielsen, Medini Steger, Nicolai J. Wewer Albrechtsen, Matthias Mann

**Affiliations:** 1 Department of Proteomics and Signal Transduction, Max Planck Institute of Biochemistry, Martinsried, Germany; 2 NNF Center for Protein Research, Faculty of Health and Medical Sciences, University of Copenhagen, Copenhagen, Denmark; 3 BioInnovation Institute, OmicVision Biosciences, Copenhagen, Denmark; 4 ions.bio GmbH, Planegg, Germany; 5 Department of Computational Biomarker Discovery, Novo Nordisk, Copenhagen, Denmark; 6 Department for Clinical Biochemistry, University Hospital Copenhagen - Bispebjerg, Copenhagen, Copenhagen, Denmark

**Keywords:** personalized medicine, clinical proteomics, mass spectrometry–based proteomic, artificial intelligence in proteomics, biomarker discovery

## Abstract

The 68th Benzon Foundation Symposium brought together leading experts to explore the integration of mass spectrometry–based proteomics and artificial intelligence to revolutionize personalized medicine. This report highlights key discussions on recent technological advances in mass spectrometry–based proteomics, including improvements in sensitivity, throughput, and data analysis. Particular emphasis was placed on plasma proteomics and its potential for biomarker discovery across various diseases. The symposium addressed critical challenges in translating proteomic discoveries to clinical practice, including standardization, regulatory considerations, and the need for robust “business cases” to motivate adoption. Promising applications were presented in areas such as cancer diagnostics, neurodegenerative diseases, and cardiovascular health. The integration of proteomics with other omics technologies and imaging methods was explored, showcasing the power of multimodal approaches in understanding complex biological systems. Artificial intelligence emerged as a crucial tool for the acquisition of large-scale proteomic datasets, extracting meaningful insights, and enhancing clinical decision-making. By fostering dialog between academic researchers, industry leaders in proteomics technology, and clinicians, the symposium illuminated potential pathways for proteomics to transform personalized medicine, advancing the cause of more precise diagnostics and targeted therapies.

## Highlights

• Mass spectrometry–based proteomics is ready to transform clinical diagnostics.
• Spatial proteomics revolutionizes disease mechanism studies.
• Population-scale plasma proteomics studies define a new era of biomarker discovery.
• Clinical proteomics requires regulatory compliance and clear medical value.
• AI transforms proteomic data acquisition, analysis, and clinical decision support.

## Graphical Abstract

**Figure:**
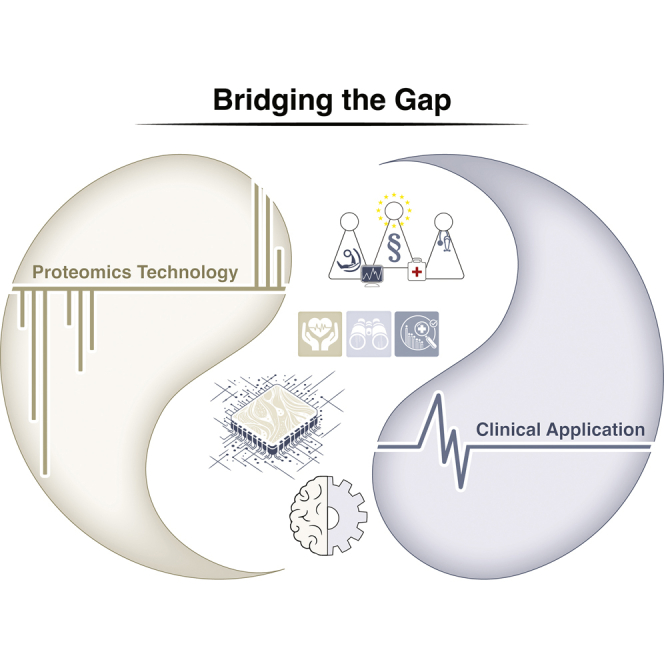

## In Brief

The 68th Benzon Symposium brought together leading experts to explore how mass spectrometry–based proteomics is transforming clinical diagnostics. Recent technological advances now enable comprehensive protein analysis at unprecedented depth, speed, and robustness. The symposium highlighted groundbreaking applications in spatial proteomics, population-scale plasma studies, and artificial intelligence–driven data analysis. Participants defined key requirements for successful clinical implementation, from regulatory compliance to medical value creation, paving the way for proteomics to revolutionize personalized medicine.

In early September 2024, the picturesque city of Copenhagen hosted the 68th Benzon Foundation Symposium, bringing together a diverse group of experts from across the globe to discuss the integration of mass spectrometry (MS)–based proteomics and artificial intelligence (AI) in personalized medicine. This gathering, titled "Integration of Mass Spectrometry-Based Proteomics and AI to Revolutionize Personalized Medicine," was a testament to the Foundation's commitment to fostering scientific dialog since the 1950s. The event was held in the iconic Radisson Collection Royal Hotel, formerly known as the SAS Hotel, a modernist masterpiece situated in the heart of the Danish capital.

Organized primarily by Matthias Mann, with support from Lars Fugger and Finn Cilius Nielsen, the symposium attracted participants from as far afield as California, Pakistan, and China. The speakers represented a broad spectrum of expertise, from cutting-edge technology developers to practitioners at leading medical centers worldwide, all united by a common goal: to explore the current status and future potential of MS-based proteomics in clinical applications.

**Fig. 1:**
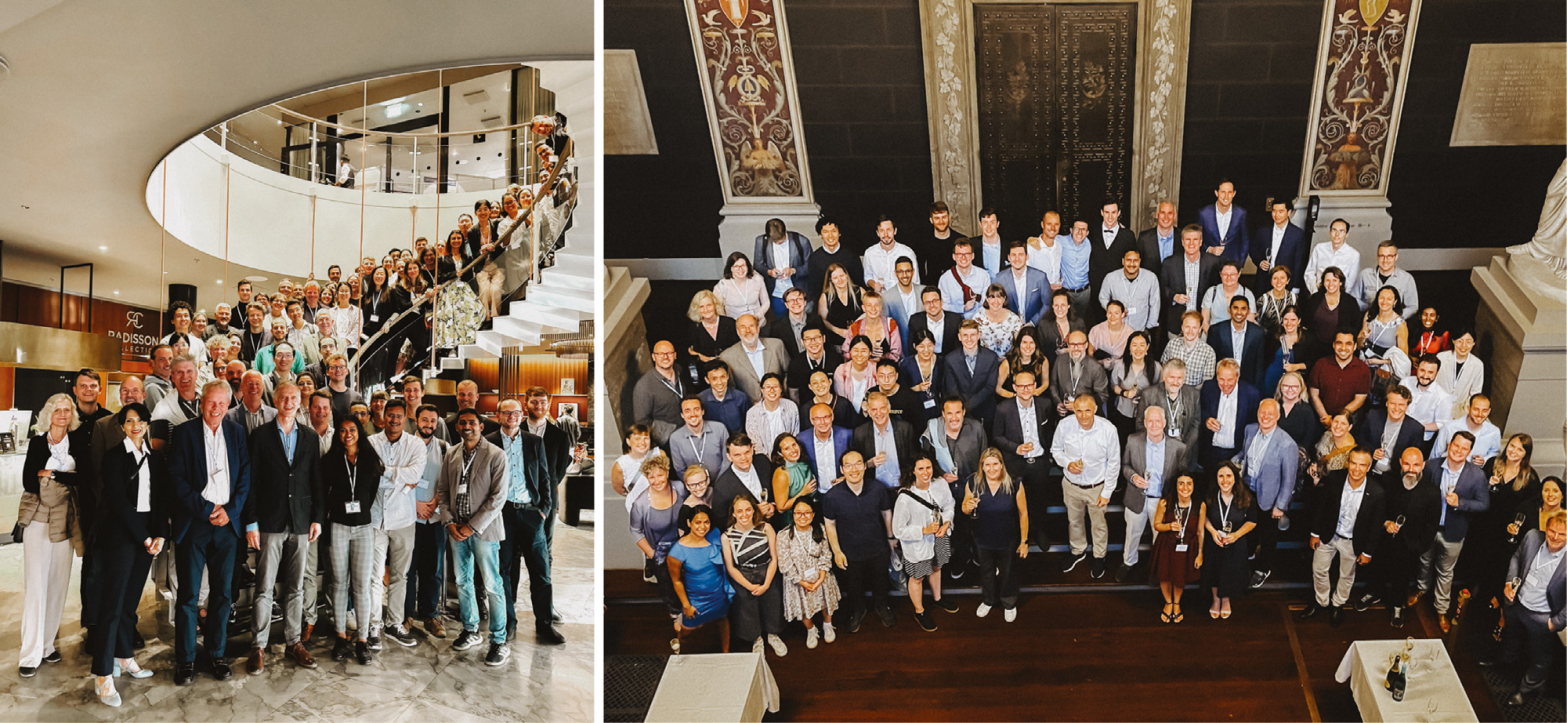
**Participants of the 68th Benzon Foundation Symposium gathered at two historic venues: the iconic Radisson Collection Royal Hotel, formerly known as the SAS Hotel (*left*) and the Ceremonial Hall of the University of Copenhagen (*right*).** Photo credits: Ashok Kumar Jayavelu, Jennifer Gillete, and Janina Albrecht.

The scientific program was complemented by a rich social agenda, including a Welcome Reception at Café Mazzolis in the historic Tivoli Gardens and a conference banquet, held in the Ceremonial Hall of the University of Copenhagen—one of the oldest universities in Northern Europe (Fig. 1).

Beyond the formal presentations and discussions, the symposium offered invaluable opportunities for informal networking, particularly beneficial for early-career scientists. These interactions fostered collaborations and sparked ideas that promise to shape the future of proteomics research and its clinical applications.

The symposium’s primary objective was twofold: to take stock of the current status of MS-based proteomics in clinical settings and to identify the obstacles hindering its widespread adoption. By bringing together technology innovators and clinicians eager to harness the power of proteomics, the symposium created a unique forum for addressing these challenges head-on.

As participants gathered in this stimulating environment, the stage was set for four days of intense scientific exchange, laying the groundwork for the next steps in revolutionizing personalized medicine through the power of proteomics and AI. This report captures the essence of these discussions, highlighting key insights and charting the path forward for this rapidly evolving field. We present perspectives from leading experts on the integration of proteomics and AI in precision medicine, covering technological breakthroughs, clinical applications, and translational challenges in bringing proteomics to patient care. We also highlight critical perspectives on the future of clinical proteomics, its potential to transform diagnostics and treatment, and the hurdles to be overcome. While not intended as a comprehensive review, we aim to embed the discussions in the wider field and provide references to the interested reader.

## The Technological Revolution Enabling Clinical Proteomics

Over the past decade, proteomics has undergone another remarkable transformation. MS-based workflows have evolved from identifying hundreds of proteins from milligram sample amounts to quantifying nearly the entire expressed proteome from microgram or even nanogram amounts of material. This leap forward is not merely incremental but represents a fundamental shift in our ability to probe the human proteome at a scale and speed previously unimaginable (1, 2).

This Benzon Foundation Symposium shed light on several key technological breakthroughs currently driving this revolution. At the forefront are significant advances in sample preparation techniques, a critical step in the proteomics workflow. Innovations and automation in this area have dramatically enhanced the depth, reproducibility, and throughput of proteomic analyses, paving the way for more robust and clinically relevant assays (3).

Instrumentation has kept pace with these developments, with instruments like the latest Bruker timsTOFs (4, 5, 6) and the Orbitrap Astral from Thermo Fisher Scientific (7, 8) featuring prominently in the talks. A notable highlight was the Stellar mass spectrometer, introduced by Stevan Horning of Thermo Fisher Scientific. This targeted instrument is similar to a triple quadrupole, but with the last quadrupole replaced by an ultra-fast linear ion trap (9, 10). This hardware innovation promises complete MS2 spectra instead of just selected transitions and is supported by advanced software for assay development and analysis, such as the PRM conductor, which converts discovery data into targeted parallel reaction monitoring assays. The emphasis on improved reproducibility, throughput, simplicity, sensitivity, and cost-effectiveness reflects the field's growing focus on clinical translation.

Symposium discussions touched on the industry-wide challenges of meeting regulatory requirements such as CE *In Vitro* Diagnostic Medical Device Regulation (IVDR) certification, ISO 13485 quality management standards, and Food and Drug Administration 510(k) clearance for clinical use. Many academic laboratories are not aware of these regulatory hurdles and the effort and time to overcome them. These regulatory hurdles, along with the need for user-friendly robust systems suitable for clinical environments, underscore the complex balance between technological advances and implementation of proteomics experiments in routine clinical tests. While we are still some ways from the ideal blood analyzer (11), the technology is rapidly moving in this direction.

Data acquisition and analysis methods for discovery proteomics have also seen significant improvements, complementing advances in sample preparation and instrumentation. The widespread adoption of data-independent acquisition (DIA) techniques has transformed proteomics workflows (12, 13, 14, 15). Alongside DIA, sophisticated software tools such as DIA-NN and Spectronaut have become pivotal in extracting meaningful insights from the complex spectra generated (16, 17, 18). AlphaDIA, introduced by the Mann group, represents a next step in open-source proteomics software packages (19, 20). This tool utilizes end-to-end transfer learning algorithms to improve peptide identification and quantification. Tiannan Guo’s group at Westlake University in China leveraged transformer-based pretrained AI models in their DIA-BERT. Such software advances will be crucial in unlocking the full potential of DIA, enabling deeper proteome coverage and more reliable quantification.

As symposium participants noted, technological advances by themselves are not sufficient to drive clinical adoption. The field now faces the challenge of translating these powerful tools into practical clinically relevant applications. This transition requires not only further refinement of technologies but also addressing issues of standardization, regulatory compliance, and integration into existing health care systems, as detailed in subsequent sections.

## Proteomics to Study Pathological Disease Mechanisms

The Benzon Foundation Symposium showcased proteomics' role in deciphering complex biological mechanisms, from cellular processes to disease pathology.

Recent methodological advances in protein labeling and quantification strategies provide deeper insights into cellular signaling pathways and regulatory mechanisms. For instance, proximity biotinylation was employed by Mara Monetti of the Memorial Sloan Kettering Cancer Center in New York to study the regulation of glucose uptake in skeletal muscle, identifying known and novel mediators of GLUT4 trafficking (21). SICyLIA (Stable Isotope Cysteine Labeling with IodoAcetamide), developed by Sara Zanivan's team at the Cancer Research UK Beatson Institute in Glasgow, offers an interesting and novel approach to understanding oxidative signaling pathways (22, 23). This method enables precise unbiased measurements of protein oxidation across the entire proteome, revealing critical insights into oxidative signaling in liver disease and cancer. Additionally, SPIED-DIA (spike-in enhanced detection in DIA), introduced by Matthias Selbach's group at the Max-Delbrück Center for Molecular Medicine Berlin, combines DIA with spike-in of stable-isotope labeled phosphopeptides to facilitate the targeted detection of particularly informative phosphorylation sites (24). This method has uncovered synergistic signaling responses to mitogen-activated protein kinase kinase inhibitors in colorectal cancer cells.

Extending beyond experimental approaches, mining the growing proteomics databases holds great promise for generating biological insights. An *in silico* reanalysis of >10,000 proteomics raw files led by Felix Meissner from the Institute of Innate Immunity of the University Hospital Bonn identified novel truncated variants of secretory proteins by performing systematic semitryptic digest searches. These previously unreported N-terminal protein variants with tissue- and cell-specific cleavage patterns reveal how proteolysis contributes to structural and functional diversity. Single-cell proteomics is starting to unveil mechanistic insights into cellular heterogeneity that were inaccessible through genomics or transcriptomics data alone. Sabrina Richter from the Fabian Theis group at the Helmholtz Centre Munich presented scProtVelo (single-cell Protein Velocity), a translation kinetics model that integrates single-cell proteomics and transcriptomics data to elucidate temporal gene expression dynamics within single cells, which was powerfully applied to the complex differentiation process of human hematopoietic stem and progenitor cells (25).

Proteomics approaches have proven instrumental in deciphering disease mechanisms across various conditions. Judith Steen from Harvard Medical School described FLEXITau for defining the post-translational modification landscape of Tau protein in Alzheimer's disease (26, 27, 28). Furthermore, the proteomics analysis of cerebrospinal fluid has yielded valuable insights into Niemann–Pick disease type C (NPC), a lysosomal storage disease that manifests as a neurodegenerative disorder. Sebastian Virreira Winter of ions.bio presented a study on cerebrospinal fluid proteomes from NPC patients, revealing significant alterations in the NPC proteome and identifying protein patterns indicative of disease progression and treatment status.

### Proteomics Advances in Cancer Research and Drug Development

Cancer biology was a central theme at the Benzon symposium, with researchers presenting innovative approaches to address critical clinical needs. Ole Nørregaard Jensen from the University of Southern Denmark is pioneering the MALDI-based MS imaging and machine learning (ML) to classify renal cancer tumor subtypes. Despite the challenges of sequencing peptides by MALDI, given its fast rate of data acquisition, it was discussed if sets of peptide masses could be sufficient for some diagnostics tasks. In addressing gynecological cancers, Tamar Geiger of the Weizmann Institute of Science highlighted the urgent demand for early ovarian cancer detection, a challenge poorly met by current CA125-based and ultrasound screening methods. Her team’s proteomic analysis of uterine lavage fluid as a proximal liquid biopsy promises to capture early events of cancer with increased specificity (29, 30).

Focusing on pediatric oncology, Ashok Kumar Jayavelu of the KiTZ-Hopp Children's Cancer Center Heidelberg turned the spotlight on treatment-resistant pediatric cancers, pointing out high relapse rates and reduced long-term survival (31). His proteomics and multiomics profiling of osteosarcoma revealed functionally relevant proteome changes, particularly in RNA processing pathways, potentially contributing to treatment resistance. Advancing immunotherapy approaches, Jennie Lill of Genentech presented a step toward a personalized cancer vaccine platform. Her team developed HLApollo, a pan-allelic, transformer-based model that integrates peptidomics and transcriptomics data to improve the prediction of peptide presentation by major histocompatibility complex I—a significant advance toward individualized neoantigen-specific therapies (32).

Michael Gillette presented work by the Clinical Proteomic Tumor Analysis Consortium (CPTAC), a flagship program of the National Cancer Institute. CPTAC aims to accelerate the understanding of the molecular basis of cancer through extensive proteogenomic tumor characterization and translational research. This approach has led to substantial contributions in understanding various cancer types (33, 34, 35, 36) and is also paving the way for precision oncology by identifying novel clinical strata and oncogenic mechanisms, proposing therapeutic vulnerabilities and candidate biomarkers. In addition, he also emphasized that efforts in precision oncology should start in an “intentional” way with a clinical question or need that is important, specific, and tractable.

The practical application of proteomics in precision oncology was further discussed by Bernhard Küster of the Technical University of Munich (37, 38). His Tumor Proteome Activity Status project is an end-to-end processing pipeline that integrates proteomics and phosphoproteomics data into a simple tumor scoring system to support clinicians with information on involved biological pathways, proteins, and phosphorylation sites. The ultimate aim of Tumor Proteome Activity Status project is to demonstrate that integrating these (phospho-) proteome-based recommendations into Molecular Tumor Boards can significantly enhance patient outcomes, paving the way for more personalized and effective cancer treatments.

A major challenge remains in delivering safer and more effective drugs suitable for a diverse global population. In his compelling keynote address, Angus Lamond of the University of Dundee emphasized the importance of recognizing human proteome diversity in health and disease, highlighting the role of genetic variation, epistasis, and proteoepistasis in shaping individual responses to treatments (39). His work with twins and induced pluripotent stem cells offers a promising approach to model population diversity in drug responses. By performing proteomics on a systematic pipeline of high-quality–induced pluripotent stem cell lines from both healthy donors and disease cohorts, Lamond's team has mapped hundreds of protein quantitative trait loci (pQTLs) and demonstrated the potential of using cell painting and MS-based proteomics to analyze mechanisms of variable drug responses (40, 41). This research not only underscores the complexity of biological systems but also points toward a future where proteomics-driven approaches could significantly improve drug development success rates and advance truly personalized and equitable medicine.

## Spatial Proteomics Meets Pathology

**Fig. 2:**
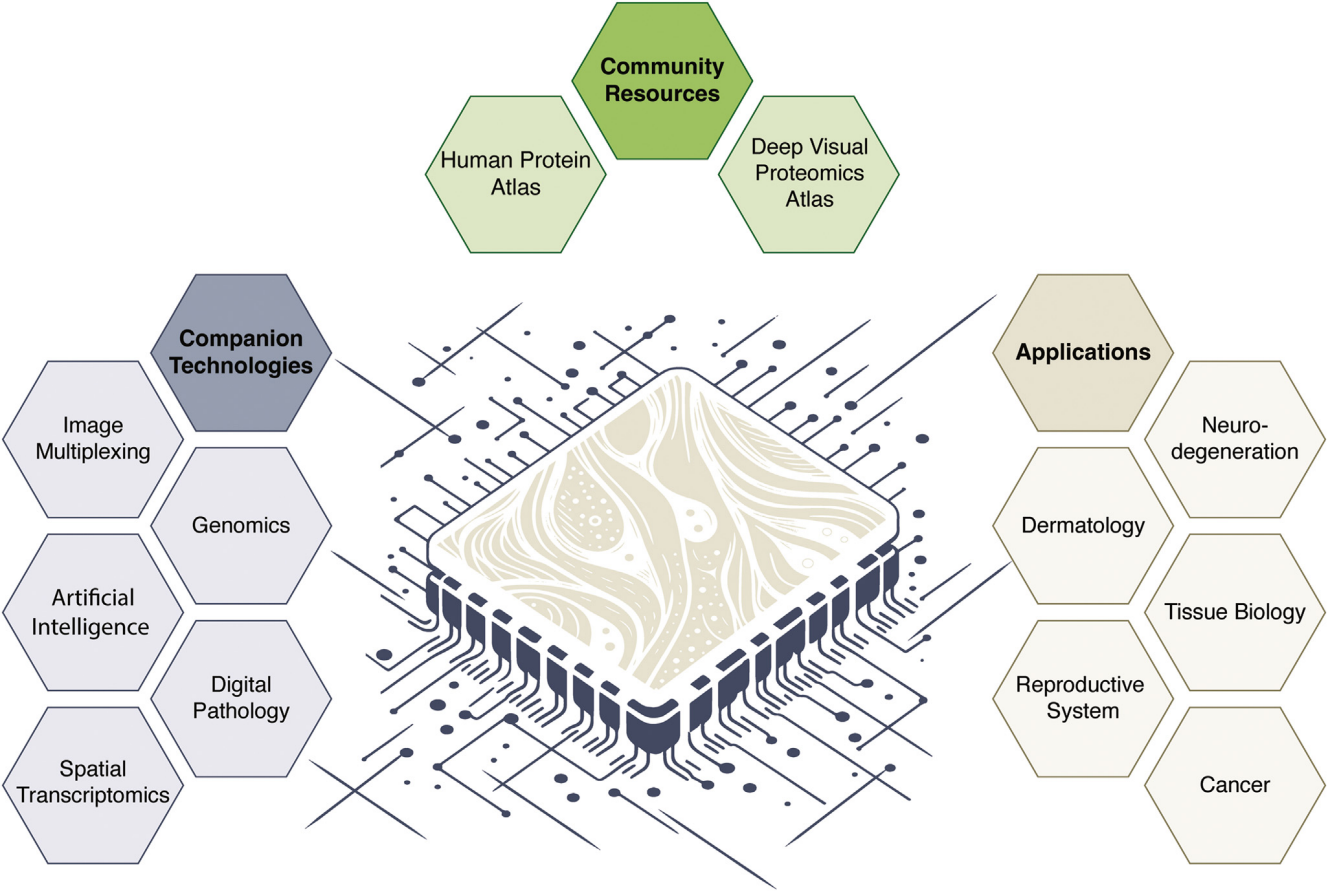
**Spatial proteomics: From tissue to single-cell resolution.** This schematic illustrates spatial proteomics technologies and applications discussed at the symposium. Spatial proteomics, depicted as a tissue section embedded in a microprocessor-inspired design, leverages companion technologies (*left*, *blue hexagons*) to study diverse clinical applications (*right*, *beige hexagons*). Community resources (*top*, *green hexagons*), such as the Human Protein Atlas and the upcoming Deep Visual Proteomics (DVP) Atlas, accelerate the field.

Spatial proteomics has emerged as a powerful approach to understand the complex organization of proteins within tissues and cells, offering unprecedented insights into biological processes and disease mechanisms (42). The symposium highlighted several groundbreaking advances in this field (Fig. 2).

A standout contribution was the keynote lecture from Mathias Uhlén, Professor at the Royal Institute of Technology and founder of the Science for Life Laboratory in Stockholm, who spectacularly described the Human Protein Atlas (HPA) (43, 44, 45, 46, 47, 48, 49, 50). Over the last decade, HPA has evolved into a cornerstone resource in mapping the human proteome across cells, tissues, and organs using antibodies. With the integration of multiplexed imaging, bulk RNA sequencing, and spatial transcriptomics, it now promises to further enhance our understanding of tissue complexity and disease-specific protein signatures. Studies on the fallopian tube and spermatogenesis showcased how these multiomics approaches can reveal intricate details of tissue-specific biology.

Complementing this work, the Mann group is developing a spatial and single-cell type MS-based map of the human body. This atlas, built upon the Deep Visual Proteomics (DVP) technology (51, 52), is designed as a community resource to push the boundaries of MS-based spatial resolution and its use cases, and there are plans to integrate it with the HPA.

The DVP approach has recently demonstrated its transformative potential in precision medicine in a case study where it discovered a successful treatment for patients with toxic epidermal necrolysis, a previously often lethal immune-mediated skin reaction (53). The versatility of DVP is further underscored by ongoing work from the Steen and Mann groups that will map proteomic changes in Alzheimer's and Parkinson's disease brains, including spatial PTM changes in alpha-synuclein. By targeting the distribution of misfolded proteins and aggregates, DVP offers new avenues for understanding disease progression and identifying potential therapeutic targets.

Integration of multiple data modalities has become a key trend in spatial proteomics. Andreas Mund, of the Medical School of the University of Copenhagen and cofounder of OmicVision Biosciences, described combining DVP with multiplexed imaging to study tumor microenvironments. This novel spatial proteomics approach revealed distinct cellular interactions in "cold" colorectal and "hot" tonsil cancers, exposing immunosuppressive macrophage-mediated barriers, T-cell behaviors, and metabolic adaptations (54). Similarly, the integration of DVP with spatial transcriptomics data by Fabian Coscia at the Max Delbrück Center Berlin in non–small cell lung cancer research highlighted the complementary nature of these techniques in unraveling the complexities of tumor microenvironments (55).

Peter Horvath from the Institute of Biochemistry in Szeged presented a comprehensive computational pipeline for single cell–based large-scale microscopy experiments, introducing novel methods for image correction, segmentation, and phenotype classification using advanced ML techniques (56, 57, 58). He described the Advanced Cell Classifier for efficient phenotype identification and proposed a multiparametric regression to analyze continuous biological phenomena. They demonstrated the power of this AI-driven digital pathology in identifying melanoma recurrence risk markers using DVP (59).

As the field moves from research to practical applications, efforts to commercialize and standardize spatial proteomics techniques are gaining momentum. Lisa Schweizer's presentation on OmicVision's development of foundation models integrating ML with DVP for clinical diagnostics exemplifies this trend toward translational research.

Emerging technological innovations continue to drive the field forward. Tiannan Guo demonstrated the potential of the ProteomEx technique, which expands tissue samples sixfold (60, 61) thereby facilitating cellular and subcellular sample collection. Generating profiles across entire tissue sections, Roman Fischer of Oxford University described "Deep Topographic Proteomics" *via* grid-based laser microdissection, enabling both large-scale, low-resolution, and small-scale, high-resolution spatial analysis (62).

## Plasma Proteomics: Mining the Circulatory Proteome

Plasma proteomics stands at the forefront of clinical translation, offering valuable insights into human health through minimally invasive sampling, especially as “single drop” amounts are now sufficient (63). Recent technological advances have set the stage for a molecular rethinking of how we approach plasma analysis (64).

Large-scale plasma studies capable of analyzing hundreds to thousands of samples per week at a fraction of previous costs are taking center stage, as exemplified by efforts of the Mann group who are analyzing 50,000 plasma samples in a single project. This paradigm shift is driven by innovations in MS technology and sample preparation techniques. Depletion methods such as the perchloric acid workflow (65), nanoparticle-based enrichment techniques such as Tiannan Guo's OmniProt beads, Seer’s Proteograph workflow (66) presented by Michael Wierer, and PreOmics’ ENRICHplus kit and Evosep's MagNet EV-enrichment workflow (based on (67)) have significantly enhanced proteome coverage and throughput while requiring only low sample volumes. In addition to robust and cost-effective sample preparation workflows, initial sample quality is critical in unlocking the full potential of plasma proteomics. High-quality samples with comprehensive clinical data, standardized collection, and processing protocols are essential to ensure consistency across large or small cohorts and to avoid biases in downstream analysis (68, 69). Jochen Schwenk at the Royal Institute of Technology Stockholm emphasized the importance of longitudinal and remote sampling strategies to effectively capture proteome dynamics (70, 71, 72, 73). This approach, combined with embracing population-wide heterogeneity in age, sex, body mass index, genetics, medication, and health history, marks a starting point for understanding individual variation rather than viewing it merely as a confounding factor.

The symposium highlighted several major initiatives in plasma proteomics. The Human Disease Blood Resource, presented by Mathias Uhlén, is an effort to create an open-access resource for precision medicine on a population scale. By utilizing antibody-based technologies, such as those commercialized by Olink (74) alongside targeted MS-based proteomics, protein levels were mapped in nearly 10,000 individuals across healthy individuals and 60 diseases. This resource, set for release in October 2024, also integrates data from major initiatives such as the CPTAC and the UK Biobank. It reveals the complex interplay between proteins, disease traits, and individual variability as exemplified by the case of fibroblast growth factor 21. Although initially considered a promising biomarker for pancreatic cancer, it is found elevated across multiple diseases. This underscored the limitations of single-marker and single disease approaches and the need for pan-disease biomarker panels.

Lili Niu, formerly of the Mann group and now at Novo Nordisk, discussed how plasma proteomics can be used to map pQTLs. She pointed out that in pQTL studies using affinity-based proteomic techniques, the most frequently used technique to date, up to a third of the reported pQTLs might be incorrectly identified because of epitope-modifying variants (75, 76, 77). By leveraging peptide-level data, artifactual pQTLs can be detected and eliminated, as powerfully demonstrated in her MS-based study in children and adolescents, discovering hundreds of extremely reproducible pQTLs across clinical studies that were not picked up by affinity-based methods (78). Her findings emphasize not only the necessity for comprehensive coverage of the plasma proteome but also raise important questions regarding quantification reproducibility and fidelity, particularly when employing protein enrichment techniques.

Precise protein quantification emerged as a central theme. In collaboration with Absea Biotechnology, the Mann group presented the use of heavy N15-labeled recombinant protein standards, aiming to provide a community-wide resource for absolute protein quantification (9). Further standardization efforts, such as the Charité Open Peptide Standard for Plasma Proteomics introduced by Markus Ralser at the Charitè University Medicine in Berlin (79) and standardized recombinant protein panels from ProteomeEdge, are addressing key challenges in translating plasma proteomics to clinical applications (80, 81, 82). Jennifer van Eyk from Cedar Sinai Medical Center highlighted the stringent requirements for clinical assays, such as maintaining a CV of less than 5% for technical replicates. Her approach involves running quality controls for every measured sample and performing regular system suitability tests to ensure consistent performance. She also introduced tools such as TEAQ for peptide selection (83) and BIRCH for batch effect correction (84), underscoring the community’s efforts to develop robust and clinic-ready methodologies.

Symposium participants agreed that plasma proteomics needs to develop robust and reproducible assays capable of detecting clinically relevant protein changes across large populations. As these techniques mature and advance toward clinical application, challenges remain in standardization, validation, and regulatory compliance. Nevertheless, the field is poised to transform clinical diagnostics and personalized health care, unlocking the full potential of the plasma proteome as a source of diagnostic and prognostic biomarkers.

## From Bench to Bedside: Navigating the Translational Landscape

**Fig. 3:**
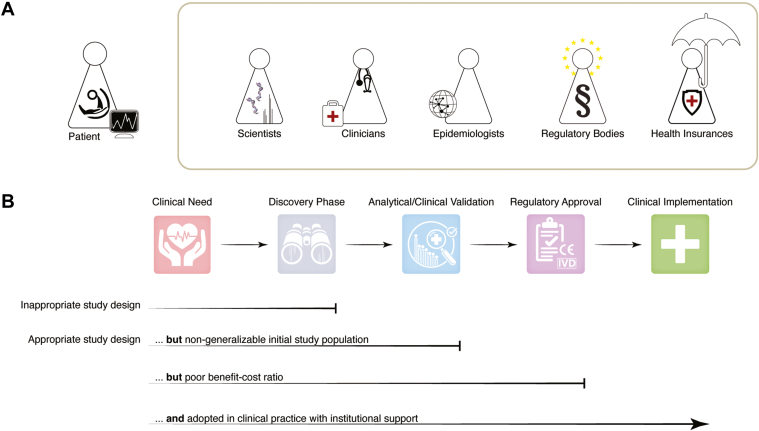
**Biomarker-discovery pipeline: From concept to clinic.***A*, schematic representation of key stakeholders in the clinical proteomics landscape, including patients, health care providers, epidemiologist, insurers, regulatory bodies, and scientists, all of whom influence the translation process. *B*, the five major stages of biomarker development (*top*) from identifying clinical need through to implementation with common roadblocks at different prevalence and scale scenarios (*bottom*). A common barrier is inappropriate study design, which prevents identification of meaningful biomarker candidates from the onset. Even with an appropriate study design, biomarker development can fail at multiple points: discovery-phase findings may not validate in broader populations; validated biomarker may lack sufficient cost–benefit advantages over existing methods. Beyond regulatory approval, successful translation into clinical practice requires careful consideration of study design, population representation, health care impact, and institutional support.

The translation of biomarker candidates is a complex and multistage process ranging from initial discovery through analytical and clinical validation, regulatory approval, and finally clinical implementation. Over the past decade, only two biomarker candidates have been approved by the Food and Drug Administration annually, underscoring the significant challenges the community has faced in study design, standardization, and validation efforts. However, the advent of population-wide studies enabled by recent breakthroughs in analytical capabilities has created a unique opportunity to overcome this “reproducibility crisis.” The symposium aimed to bring together all key stakeholder—clinicians, epidemiologists, cell biologists, proteomics specialists, bioinformaticians, and industry partners—to facilitate the translation of proteomics results and biomarker candidates into clinical practice (Fig. 3).

### Defining the Clinical Need

The successful translation of proteomics experiments into clinical practice hinges on addressing unmet clinical needs. Nicolai Wewer Albrechtsen at Bispebjerg Hospital and University of Copenhagen underscored this point, noting that 72% of existing biomarkers have no impact on patient outcomes. To bridge this gap, Michael Gillette emphasized the need for "intentional" efforts in precision medicine as already mentioned above. These efforts should consider several key factors: (i) Expected clinical impact, both for individual patients and affected populations. (ii) Type of test required (screening, diagnostic, or prognostic), (iii) Appropriate sample type (*e.g.*, blood, urine, tissue), and (iv) Resource availability for development and implementation.

The case of neuroborreliosis plasma biomarker development by Nicolai Wewer Albrechtsen illustrates these principles in action. This frequent bacterial infection of the central nervous system caused by tick bites presents a diagnostic challenge. Current methods are invasive, lack diagnostic accuracy, and result in substantial diagnostic delays. A proteomic approach to this problem directly addresses these clinical needs by offering a noninvasive, accurate, and rapid diagnostic tool. Similarly, Michael Gilette highlighted the critical need for improving risk stratification in multiple myeloma with markers predictive for progression. Current gold standard markers for multiple myeloma involve invasive and potentially inconsistent bone marrow biopsies, rendering serial sampling clinically infeasible. Plasma proteomics could offer a promising and minimally invasive alternative that could transform patient care by enabling more frequent and consistent disease monitoring, potentially capturing disease progression and improving early detection and treatment outcomes.

The successful translation of proteomics research into clinical practice requires careful consideration of study design, particularly in relation to the intended use of biomarkers. Aleksander Krag, expert in hepatology at Odense University Hospital, highlighted that the diagnostic accuracy of a biomarker can vary significantly depending on the prevalence of the disease in the studied population, a phenomenon known as the spectrum effect. This underscores the importance of tailoring biomarker development to specific clinical contexts and testing in cohorts that reflect appropriate disease prevalence (85). Complementing this aspect, Majken Karoline Jensen of the Department of public Health at the University of Copenhagen emphasized integrating epidemiological principles with proteomics-specific considerations such as standardized sample handling and robust analytical validation. By combining these approaches, researchers can increase the likelihood of developing clinically useful biomarkers that address real unmet needs, building the evidence base necessary for regulatory approval and clinical adoption.

### Regulatory Approval

Clinical biomarkers should adhere to strict criteria: they are supposed to be precisely determinable analytes based on defined test materials and determination methods, with absolute quantification in standardized units traceable to certified reference materials. These biomarkers are usually requested and reported as single analytes and provide a universally adaptable and interoperable toolbox for clinicians worldwide.

At the symposium, Daniel Teupser, of the Institute of Laboratory Medicine at the University Hospital LMU Munich, highlighted two options for introducing (plasma) proteomics into the clinical laboratory in compliance with the new EU IVDR enforced in May 2022 (86, 87): (i) the use of certified *In Vitro* Diagnostic devices, which have undergone extensive clinical testing and technical documentation by the manufacturer and certification by a notified body. (ii) Health care institutions developing their own "devices manufactured and used only within health institutions" (Article 5, EU IVDR), referred to as laboratory-developed tests (LDTs). While IVDR recognizes the importance of in-house procedures, particularly for rare diseases and as drivers of innovation in laboratory medicine, these LDTs are subject to increasingly stringent regulations, such as an appropriate quality management system, ISO 15189 compliance, and postimplementation monitoring.

Successful navigation of these regulatory hurdles requires proteomics researchers to carefully consider clinical needs, technical capabilities, and regulatory requirements at each stage of development. A prime example of this is Nicolai Wewer Albrechtsen’s ongoing implementation of MS-based proteomic analysis in Copenhagen hospitals for liver diagnostics and benign hematology. However, challenges remain in translating these successes to other countries, as each has its own legislative frameworks and reimbursement structures.

### Standardization and Quality Control in Clinical Proteomics

Quality assurance remains a cornerstone of clinical implementation, with regulations varying at national levels. According to Daniel Teupser, most laboratories adhere to ISO 15189 standards for medical laboratory accreditation. For example, in Germany, the Federal Medical Council (Rili-BÄK) provides precise specifications for quality management. These include internal quality controls at the start and end of each measuring period or at least every 16 h for continuous tests and external quality controls every 3 to 6 months as part of ring trials.

A key concept in this quality assurance framework is metrological traceability, ensuring full traceability of laboratory results. This ensures that measurement results can be related to a reference through a documented unbroken chain of calibrations, each contributing to the measurement uncertainty. Interlaboratory comparisons further reinforce this standardization, with methods traceable to certified standards, starting from SI units through calibrators, thus ensuring global comparability of results.

Recognizing these challenges, the proteomics community has emphasized the critical importance of standardization and quality control in translating proteomic discoveries to clinical practice. Several speakers at the symposium, including Markus Ralser, Jennifer van Eyk, and Matthias Mann, presented initiatives aimed at establishing standardized workflows and quality control measures. These efforts are crucial for overcoming standardization challenges and building confidence in proteomic biomarkers.

### Education and Training Needs for Clinical Staff

The need for education and training of clinical staff emerged as another crucial factor for the successful adoption of proteomics in health care. Robust systems that can operate 24/7 with minimal downtime need to be user-friendly and must require minimal intervention from clinical staff. Equally important is the translation of the proteomic test results into clinically relevant metrics. In this context, Aleksander Krag pointed out that traditional metrics like receiver operating characteristic curve, sensitivity, and specificity may be less relevant in clinical settings. Instead, clinicians require practical insights into a test's real-world performance, including rates of correct classification and the implications of false positives and negatives.

Participants agreed that bridging the knowledge gap between proteomics researchers and clinicians is essential for ensuring that complex proteomic data can be effectively interpreted and applied in clinical decision-making. However, they also recognized that this educational challenge extends beyond health care providers to encompass patients, policymakers, and other stakeholders in the health care ecosystem.

The discussion underscored the importance of developing comprehensive educational programs and user-friendly interfaces to facilitate the integration of proteomics into routine clinical practice. This multifaceted approach to education and training was seen as critical for realizing the full potential of proteomics in personalized medicine.

## Building the Business Case for Proteomics in the Clinic

As academic researchers in the field of proteomics, we often find ourselves pondering a crucial question: Why are proteomics or other omics not more widely used in clinical settings already? The answer, as illuminated by discussions at the symposium, is multifaceted and rooted in both practical and economic considerations.

The economic landscape of health care implementation poses significant challenges. As Daniel Teupser highlighted, current costs for basic parameters in clinical chemistry start from mere cents for common analytes and do not exceed several euros for most protein tests. While proteomics offers a far wider spectrum of information than these established tests, it must justify its place and added value in this spectrum. The initial investment required for proteomics platforms in clinical settings remains substantial, necessitating a compelling case for long-term value.

However, the potential for cost savings through early diagnosis and personalized treatment presents a powerful counterargument. Proteomics holds the promise of identifying diseases at their earliest stages and tailoring treatments to individual patients, potentially reducing overall health care costs. Nicolai Wewer Albrechtsen's observation that some proteomic analyses are already cost-competitive with existing tests like B12 vitamin deficiency screening suggests that this future may be closer than we think.

Besides the need for cost-effectiveness, proteomics must also contend with the turnaround time of existing technologies of a few minutes for traditional immunoassays and up to 20 min for clinical chemistry analyzers. Almost half of all clinicians consider the time needed to obtain the test result as paramount in their clinical routine, given the urgency of their decisions (88). Proteomic approaches should strive to match these benchmarks to meet clinical needs.

A significant hurdle in the widespread adoption of clinical proteomics lies in navigating the complex landscape of patient consent and data protection (89). While essential for patient rights, these safeguards can impede the implementation of new technologies. Daniel Teupser proposed and is in the process of implementing a "broad informed consent," a national consent form that enables data flow into research spaces after double pseudonymization, allowing interoperability between clinical care and research.

According to Michael Roehrl of the Beth Israel Deaconess Dana-Farber Cancer collaboration and Harvard Medical School, a fully integrated oncology campus breaking ground in Q4 2024 is going to be a significant advance in supporting clinicians with multimodal data for cancer diagnostics, therapy response monitoring, and early resistance and recurrence detection. As we are in a “postgenomic era,” of Precision Pathology, it is becoming increasingly important to functionally characterize human diseases by integrating advanced multiomic molecular diagnostics such as proteomics and metabolomics, “living assays” including organoids and patient-derived xenografts, and computational diagnostics. An important step is to enable automated data extraction from electronic medical records, that is, patient specific (such as age, sex, ethnicity, comorbidities) and preanalytical data (fresh sample handling, sample processing), as part of LDT validation and routine diagnostic workflows, making biospecimen science an integral part of diagnostics. A major unmet need in multiomic precision medicine is the ability to computationally integrate multimodal quantitative data from patients (*e.g.*, at DNA, RNA, protein, and metabolite levels) with digital pathology and radiographic imaging to generate actionable clinical guidance for patient treatment. Advances in ML–AI and other mathematical modeling promise to be powerful tools for integrated molecular medicine.

The symposium also addressed the challenges of implementing clinical proteomics in low- and middle-income countries, as highlighted by the presentation on the Alliance for Maternal and Newborn Health Improvement (AMANHI) by Fyezah Jehan at the Aga Khan University in Pakistan. The AMANHI initiative aims to create the best characterized cohort of pregnant women and their babies in communities in sub-Saharan Africa and South Asia. Despite infrastructure limitations and resource constraints, innovative solutions, such as mobile laboratories and solar-powered facilities, have enabled the biorepository in Pakistan to collect over 10,000 samples. In collaboration with the Multi-omics for Mothers & Infants Consortium (MOMI), these biorepositories have been leveraged to engage in a deep omics effort to develop preventive interventions and predictors for critical adverse pregnancy outcomes.

While this effort demonstrates the feasibility of large-scale proteomics studies in resource-limited settings, translating these research findings into clinical applications remains a significant hurdle. Successful transition requires not only technological adaptations but also capacity building, sustainable funding strategies, and supportive policies. Jehan’s work underscores the need for global collaboration and knowledge exchange to ensure that clinical proteomics reaches all populations, regardless of economic status.

## AI: The Next Frontier in Proteomics

**Fig. 4:**
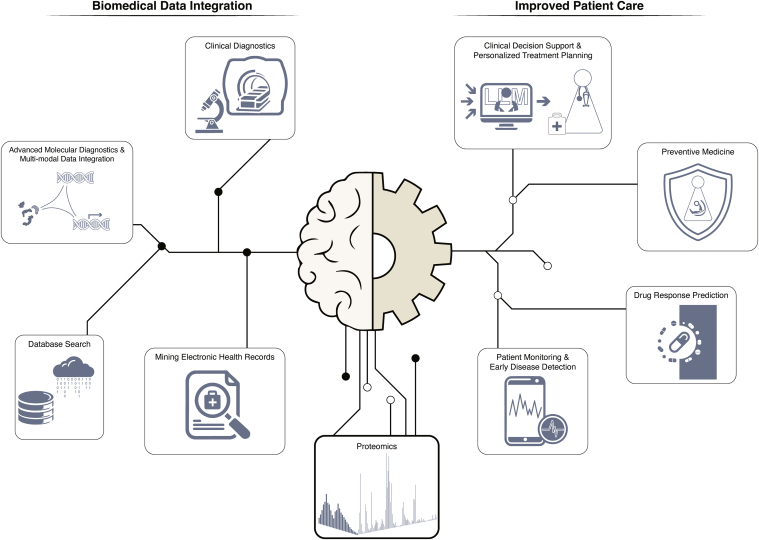
**Artificial intelligence (AI) in precision medicine: From biomedical data integration to improved patient care.** The central brain-gear motif represents AI’s dual role in processing biomedical data (*left*) and improving patient care (*right*). On the data integration side, AI integrates proteomics data with advanced molecular diagnostics, clinical diagnostics, database searches, and electronic health records. This comprehensive analysis drives improved patient care through large language model (LLM)–assisted clinical decision support, patient monitoring, drug response prediction, and preventive medicine approaches.

As proteomics technologies generate increasingly vast and complex datasets, AI and ML approaches are becoming indispensable for extracting meaningful insights and translating them into actionable clinical information (3, 15). This transformative synergy between AI and proteomics was a central theme of the symposium, reflecting its pivotal role in advancing biomedical research and precision medicine (Fig. 4).

Building on AI-driven software tools mentioned earlier, the symposium explored how to uncover subtle proteomic patterns that were previously indiscernible, for instance, by using AI-based proteomics subtyping algorithms such as REAL-XType presented by Cheng Chang. Nima Aghaeepour's work on predicting future morbidities in maternal and child health exemplified how ML algorithms can identify subtle patterns in complex electronic health record datasets. This not only aids interventions but also opens new possibilities for preventive medicine and personalized health care (90, 91). Such capabilities are opening new avenues for early disease detection, biomarker discovery, and the development of personalized treatment strategies, underscoring AI's potential to reshape the landscape of clinical proteomics.

The synergy between AI and imaging technologies was another highlight, with Peter Horvath's presentation demonstrating how ML methods in image analysis can complement proteomic data, offering a more comprehensive understanding of biological systems. This convergence of AI, imaging, and proteomics, which is also elaborated on in the aforementioned spatial proteomics section, is providing unprecedented insights into cellular heterogeneity and tissue microenvironments. These advancements hold significant implications for disease research and drug development.

However, the symposium also addressed the challenges of implementing AI solutions in clinical settings. Issues of data quality, the need for large, diverse datasets for training AI models, and the importance of interpretable AI for clinical decision-making were all acknowledged as critical areas for ongoing research and development. Cheng Chang's presentation on the π-HuB project, a multibillion-dollar, 30-year Chinese initiative in proteomics, demonstrated that large-scale collaborations and data-sharing initiatives would be crucial in overcoming these hurdles. One aim of the π-HuB project is the generation of a digital twin—computer simulations of patients—that can be used to predict molecular changes upon diseases, treatments, or other perturbations.

Looking forward, the potential of AI in proteomics extends even further, particularly with the advent of large language models (LLMs), capable of mining vast amounts of biomedical literature and electronic health records (92, 93). By integrating this information with multiomics data, LLMs could provide a more holistic view of biological systems and patient health, leading to more accurate predictions of disease risk and optimal treatment strategies. Crucially, LLMs have the potential not only to support clinical decision-making but also to enhance clinicians' understanding by providing clear and contextualized explanations of their recommendations. This interpretability is key to building trust and facilitating the adoption of AI-driven insights in clinical settings.

As the field moves toward clinical applications, there is a growing recognition of the need for standardized and reproducible AI-driven analysis pipelines that can meet regulatory requirements. The convergence of advanced AI technologies with ever-improving proteomic techniques promises to usher in a new era of biological understanding and precision medicine, making the collaboration between AI experts, proteomics researchers, and clinicians more vital than ever.

## Conclusion and Outlook

The 68th Benzon Foundation Symposium has illuminated the transformative potential of integrating MS-based proteomics and AI in personalized medicine. It is clear that the field is poised for a paradigm shift in how we approach health and disease.

The convergence of technological advancements in proteomics with the analytical power of AI is opening new frontiers in our understanding of biological systems. From unraveling complex cellular processes to mapping protein landscapes in tissues, these tools are poised to provide unprecedented insights into disease mechanisms. The ability to analyze the proteome at a population scale, as demonstrated by large-scale plasma studies, promises to revolutionize our approach to biomarker discovery and validation. However, the path from scientific breakthrough to clinical implementation remains long and challenging. The symposium underscored the critical need for addressing unmet clinical needs, navigating complex regulatory landscapes, and ensuring robust standardization and quality control. The economic considerations and practical challenges of integrating these technologies into existing health care systems cannot be overlooked.

Looking ahead, the success of clinical proteomics will depend on several key actions:

1. Develop standardized protocols for sample collection, processing, and analysis to ensure reproducibility across different laboratories and health care settings.
2. Establish international collaborations to create large-scale and diverse proteomic datasets that represent global populations.
3. Invest in user-friendly software tools that can translate complex proteomic data into actionable clinical insights.
4. Create targeted educational programs for health care professionals to bridge the knowledge gap between proteomics research and clinical practice with special attention to the needs of smaller laboratories.
5. Engage with regulatory bodies to develop appropriate frameworks for the validation and approval of proteomic-based diagnostic tools.
6. Foster partnerships between academia, industry, and health care providers to accelerate the translation of proteomic discoveries into clinical applications.

The potential impact of these advancements extends beyond individual patient care. As we develop the capability to analyze proteomes across diverse populations, including in resource-limited settings, we open the door to a more inclusive and comprehensive understanding of health and disease.

The collaboration between proteomics researchers, AI experts, clinicians, and industry partners will be more crucial than ever. The field should actively promote interdisciplinary research and create platforms for ongoing dialog between these stakeholders. The future of health care, shaped by these advancements, promises more precise diagnostics, targeted therapies, and ultimately, better outcomes for patients worldwide. Clearly, we can now accelerate the integration of proteomics into clinical practice and unlock its full potential for improving global health.

## Conflict of interest

A. M. is a part-time employee and L. S. a full-time employee of OmicVision Bioscience. L. N. is an employee of Novo Nordisk, and P. E. G. is a founder of ions.bio GmbH. M. M. is an indirect shareholder of Evosep and OmicVision Biosciences. All other authors declare no competing interests.
